# Commentary: Stimulation of the Posterior Cingulate Cortex Impairs Episodic Memory Encoding

**DOI:** 10.3389/fnhum.2020.00334

**Published:** 2020-08-28

**Authors:** Marie-Lucie Read, Rikki Lissaman

**Affiliations:** Cardiff University Brain Research Imaging Centre, School of Psychology, Cardiff University, Cardiff, United Kingdom

**Keywords:** posterior cingulate cortex, episodic memory, encoding, deep brain stimulation, hippocampus

The role of the posterior cingulate cortex (PCC) in memory is debated within human cognitive neuroscience. Recent proposals posit that the PCC is a component of a large-scale cortico-hippocampal network that supports episodic sequencing and recollection, named the posterior medial (PM) network (Ranganath and Ritchey, 2012; Inhoff and Ranganath, 2017). Attempts to deconstruct the PM network are in their infancy (Ritchey and Cooper, 2020), but it has been shown that event-specific reactivation in the PCC correlates with recall of episodic details (Bird et al., 2015). This work suggests that the PCC has an active role in the consolidation of episodic memories. However, numerous fMRI studies have also observed that successful encoding is associated with greater PCC *deactivation* (for review, see Huijbers et al., 2012). This pattern—part of the “encoding/retrieval flip” phenomenon (Daselaar et al., 2009)—instead implies that attenuation of PCC activity during encoding might facilitate memory.

In a novel study combining deep brain stimulation (DBS) and stereotactic encephalography in humans, Natu et al. (2019) explored whether PCC stimulation (~100 Hz)—assumed to be inhibitory—would improve memory and modulate hippocampal activity, if applied during the encoding-phase of a verbal free-recall task. While stimulation did modulate hippocampal gamma and theta oscillations, it also led to a mild behavioral impairment, primarily driven by poorer recall for early items in a series (i.e., reduced primacy effect). Subsequent analyses indicated that the hippocampal modulations, particularly in the low gamma band, correlated with memory disruption. The authors concluded that their findings imply a causal role for the PCC in episodic encoding.

Although this work makes an important and valuable contribution to our understanding of the role of the PCC, there is scope for further investigation. In this commentary, we examine the extent to which the conclusions are supported, with the aim of raising broader questions for the field. We draw on various methodological and theoretical considerations, highlighting potential avenues for future research.

## Experimental Controls

DBS confers a unique opportunity to selectively stimulate brain regions and observe the behavioral results, affording a level of causal inference unmatched by other methods used in human neuroscience (Poldrack and Farah, 2015). In this regard, the use of DBS is a strength of Natu et al. (2019) study. However, as the authors did not examine the effect of stimulation on a control task, or in a control region, it remains possible that the observed effects were neither specific to the PCC nor episodic encoding. From a process-based view, it is plausible that stimulation disrupted attentional processes rather than memory processes. It is understood that the allocation of attention contributes to the primacy effect (e.g., Brown et al., 2000), and the PCC has been proposed to play an important role in controlling attentional focus (Leech and Sharp, 2014). To reject such explanations and demonstrate a specific causal role for the PCC in episodic encoding, the inclusion of control tasks with little episodic-mnemonic demand, such as the spatial-cueing task, would be beneficial.

## Mechanism(s) of DBS

Despite its widespread clinical use, the mechanism(s) of DBS remain elusive (Chiken and Nambu, 2016). Contemporary research suggests that DBS acts through multiple mechanisms rather than simple local excitatory and/or inhibitory mechanisms (Ashkan et al., 2017). Accordingly, predicting whether stimulation will have a net excitatory or inhibitory effect, and the subsequent impact on behavior, is challenging. Moreover, research using closed-loop stimulation—a system in which stimulation is determined by recorded brain signals rather than fixed parameters—has shown that the effect of stimulation on episodic memory is state-dependent; it depends on the timing of stimulation relative to the brain's encoding state (Ezzyat et al., 2017, 2018). Therefore, without knowing the effect of stimulation, interpretation is challenging.

## Stimulation Parameters

Studies using DBS to examine memory processes often differ in sample size, amplitude and frequency of stimulation, and memory task (Suthana et al., 2018). Although all these factors could influence the results, differences in stimulation parameters (e.g., amplitude and frequency) are perhaps the most consequential. This is problematic for the authors' claim that the PCC's role in encoding is separate from that of the hippocampus, as it is primarily based on comparisons with a study that applied a different frequency of stimulation (~50 Hz) to the hippocampus/entorhinal cortex (Goyal et al., 2018). They describe that both studies observed a stimulation-related effect on primacy but that PCC stimulation increased temporal-clustering (i.e., the tendency to cluster recalled items based on their proximity in the encoding-phase) whereas hippocampus/entorhinal cortex stimulation decreased temporal-clustering. However, due to the different stimulation frequencies applied, it is difficult to draw such conclusions. Research that systematically examines the effect(s) of stimulation parameters on memory is necessary to facilitate concrete claims of causality in DBS memory research.

## Electrode Localization

When describing the electrode locations, Natu et al. (2019, p. 7,175) state that, “all electrodes were targeted to the retrosplenial region of the PCC, using the splenium of the corpus callosum as a landmark.” Given that this region comprised Brodmann areas 26, 29, 30, and the ventral portion of area 23, the stimulated region actually included two areas of cortex: PCC and retrosplenial cortex (Vogt, 2009). This complicates interpretation, as the PCC and retrosplenial cortex are both components of the PM network and may support different representations and/or processes (Ritchey and Cooper, 2020). While we appreciate the challenge of selectively stimulating these regions *in vivo*, it is important to note that the findings may result from combined PCC/retrosplenial cortex stimulation.

## Conclusion

Numerous fMRI studies implicate the PCC in memory, although its exact role remains undetermined. Here, we critically reviewed the findings of (Natu et al., 2019) study, which used DBS to attenuate PCC activity during encoding. Their observation that stimulation impaired recall *prima facie* suggests that the PCC actively supports encoding, a finding that appears to stand in contrast to predictions based on the “encoding/retrieval flip.” However, there are methodological and theoretical considerations that hinder this conclusion, and call for further investigation. Through this commentary, we hope to call attention to this fascinating topic and highlight important considerations for future memory research using DBS.

## Author Contributions

All authors listed have made a substantial, direct and intellectual contribution to the work, and approved it for publication.

## Conflict of Interest

The authors declare that the research was conducted in the absence of any commercial or financial relationships that could be construed as a potential conflict of interest.
